# Multiple Roles of Pitx2 in Cardiac Development and Disease

**DOI:** 10.3390/jcdd4040016

**Published:** 2017-10-11

**Authors:** Diego Franco, David Sedmera, Estefanía Lozano-Velasco

**Affiliations:** 1 Cardiovascular Development Group, Department of Experimental Biology, University of Jaén, Jaén 23071, Spain; 2 Institute of Anatomy, Charles University, and Institute of Physiology, Czech Academy of Sciences, Prague 128 00, Czech Republic; david.Sedmera@lf1.cuni.cz; 3 School of Biological Sciences, University of East Anglia, Norwich NR4 7TJ, UK; E.Lozano-Velasco@uea.ac.uk

**Keywords:** Pitx2, left/right signaling, congenital heart diseases, atrial fibrillation

## Abstract

Cardiac development is a complex morphogenetic process initiated as bilateral cardiogenic mesoderm is specified at both sides of the gastrulating embryo. Soon thereafter, these cardiogenic cells fuse at the embryonic midline configuring a symmetrical linear cardiac tube. Left/right bilateral asymmetry is first detected in the forming heart as the cardiac tube bends to the right, and subsequently, atrial and ventricular chambers develop. Molecular signals emanating from the node confer distinct left/right signalling pathways that ultimately lead to activation of the homeobox transcription factor Pitx2 in the left side of distinct embryonic organ anlagen, including the developing heart. Asymmetric expression of Pitx2 has therefore been reported during different cardiac developmental stages, and genetic deletion of Pitx2 provided evidence of key regulatory roles of this transcription factor during cardiogenesis and thus congenital heart diseases. More recently, impaired Pitx2 function has also been linked to arrhythmogenic processes, providing novel roles in the adult heart. In this manuscript, we provide a state-of-the-art review of the fundamental roles of Pitx2 during cardiogenesis, arrhythmogenesis and its contribution to congenital heart diseases.

## 1. Introduction to Cardiac Development

**Figure 1:**
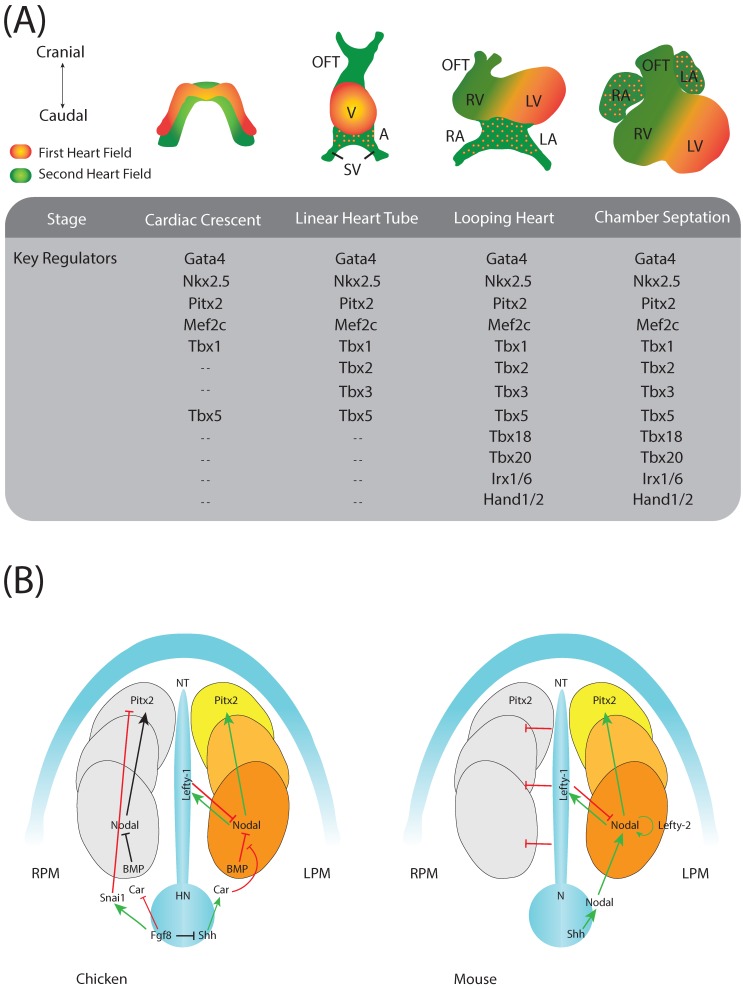
(**A**) Schematic representation of the developing heart delineating distinct developmental stages and the most prominent transcription factors involved in these morphogenetic processes. Adapted from Paige et al. [11]; (**B**) Schematic representation of the molecular left/right signalling pathway emanating from the node and leading to the activation of the homeobox transcription factor Pitx2 in the left lateral plate mesoderm (LPM) in chicken and mouse, respectively. Images adapted from Meyers & Martin [12] and Schlueter and Brand [13]. N, node; NT, notochord; HN, Hensen’s node; RPM, right lateral plate mesoderm; OFT, outflow tract; V, Ventricle; A, Atria; SV, sinus venosus; RV, right ventricle; RA, right atrium, LA, left atrium; LV, left ventricle.

Cardiac development is an intricate morphogenetic process involving distinct cell lineages. The first steps of cardiogenic formation are initiated as bilateral precardiogenic mesoderm becomes specified [1]. Soon thereafter, the bilateral cardiogenic subpopulations merge at the embryonic midline configuring a linear cardiac tube [2,3] (Figure 1). At this stage of development, the heart is symmetrical, but soon thereafter, bilateral asymmetry is established as the cardiac tube bulges on its ventral aspect and then invariably bends rightward. Prospective embryonic atrial and ventricular chambers become soon established with a common inlet, atrioventricular canal and outlet segments [1] (Figure 1). These cardiac regions are progressively derived from distinct cardiogenic cell subpopulations, the first heart field (FHF) and the second heart field (SHF) (Figure 1). As development proceeds, all cardiac chambers and segments are separated into systemic and venous components by the formation of a series of cardiac septa [4]. Inflow tract and atrial chambers are divided into right and left components by the subsequent formation of the primary and secondary atrial septa [5]. Ventricular chambers are separated by the coordinated formation of interventricular muscular and membranous septa, and distinct aortic and pulmonary trunks are divided with the formation of the outflow tract septum [6] (Figure 1). Defects or minimal delays occurring during these morphogenetic processes lead to severe cardiac congenital malformations such as double inlet left ventricle (DILV), double outlet right ventricle (DORV), atrial or atrioventricular septal defects (ASD, AVSD), among others.

While it is rather simple to ascribe embryonic right and left components to the initial symmetrical cardiac tube [7], the contribution of the mesoderm originally patterned by the left/right axis to the developing structures is far more complicated and puzzling, a puzzle that remains to be fully elucidated. Nonetheless, a clear assumption can be drawn from this evidence, i.e., disruption of left/right signalling will have an important contribution to congenital heart diseases [8,9,10].

## 2. Cardiac Regulatory Mechanisms

Over the last decade, our understanding of the molecular regulatory mechanisms underlying cardiovascular development has greatly advanced. Seminal papers reported key roles of distinct growth factors [14,15,16] and transcription factors [17] during early phases of cardiogenesis. Among those transcription factors, Nkx2.5, Mef2, Gata4 and Srf can be defined as core cardiogenic regulatory molecules [18] (Figure 1). In fact, reprogramming of skin fibroblasts into cardiomyocyte underlies the functional importance of some of these transcription factors (Mef2, Gata4) [19,20]. In addition to these “global” cardiac-enriched transcription factors, a large array of tissue-restricted transcription factors has been identified during cardiogenesis. Hand1 and Hand2 transcription factors display ventricular chamber-specific expression in the systemic and venous components [21]. Tbx1 is restricted to the arterial pole with minimal contribution to the developing myocardial component, but essential for aortic arch formation and remodelling [22]. Tbx2 is confined to discrete areas of the developing heart, such as the outflow tract and atrioventricular canal, a pattern rather similar to Tbx3, contributing thereafter to some of the prospective cardiac conduction system components [23]. Tbx5 is restricted to the systemic ventricle, as well as both atrial chambers [24,25], whereas Tbx18 is confined to a discrete subset of ventricular cardiomyocytes, as well as to the venous pole of the heart [23]. Similarly, the Iroquois family members (Irx1 to Irx6) also display tissue-specific expression and thus functional roles during cardiogenesis [26,27,28,29]. Overall, these data demonstrate a complex orchestration of temporal and tissue-specific transcriptional regulation during cardiac development.

A particular case is the homeobox transcription factor Pitx2. Pitx2 expression is already present in the developing embryo soon after gastrulation [30,31,32,33]. Pitx2 subsequently acquires a tissue-restricted pattern in the early symmetrical organ anlages, including the heart, and its expression continues to be modified as organogenesis takes place, as we will see in the following chapters.

## 3. The Homeobox Transcription Factor Pitx2

Homeobox genes encode transcription factors that bind to cognate DNA sequences through their conserved homeodomain regions [34]. Pitx1, Pitx2 and Pitx3 are three vertebrate paralogues from the Pitx family [35]. The Pitx proteins belong to the bicoid-related subclass of homeodomain proteins with the lysine residue at position nine of the third helix, being the major determinant of DNA and RNA binding specificity [36], and are highly conserved at the amino acid level at the C-terminal while significantly diverging at the N-terminal [35].

The Pitx family is crucial for the formation of several tissues. Pitx1 is involved in development and specification of a number of structures including first branchial arch derivatives, body wall and hind limb musculature [37,38,39,40], whereas Pitx2 is fundamental to brain, pituitary, facial structures and heart development [41,42,43,44,45,46]. Pitx3 is the third gene of the family, and it is central to eye and dopaminergic neurone development [47,48,49].

**Figure 2:**
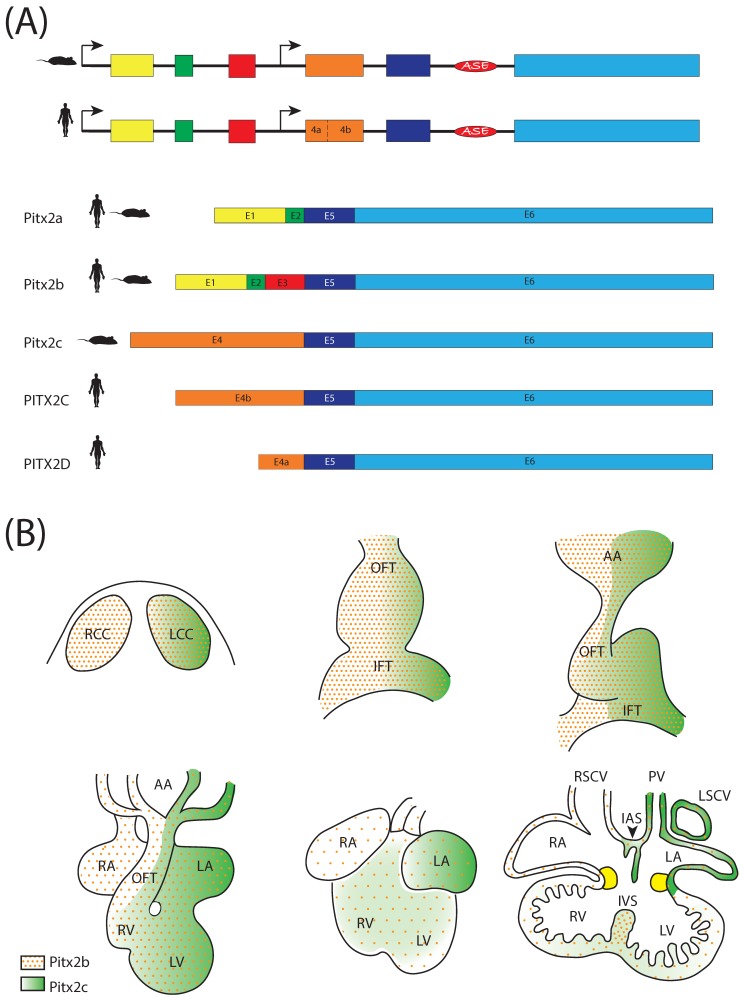
(**A**) Schematic representation of the Pitx2 genomic structure and the corresponding Pitx2 isoforms in humans and mice; (**B**) Schematic representation of the Pitx2 isoform (Pitx2b and Pitx2c) expression patterns during cardiac development.

The Pitx2 transcription factor is widely distributed in different species [42] and is encoded by the *Pitx2* gene, which is located on chromosome 3 (3G3; 3 57.84 cM) in the mouse [50]. This transcription factor displays three different isoforms, which are generated by alternative splicing with a common promoter for *Pitx2a* and *Pitx2b* located upstream of exon 1, whereas *Pitx2c* uses an alternative promoter located upstream of exon 4 and a left side-specific enhancer (ASE) upstream of exon 6 [35,43,50,51,52,53,54] (Figure 2). These three isoforms share exons 5 and 6, but differ in others; exon 3 is *Pitx2b* specific, while exon 4 is *Pitx2c* specific. Different laboratories assessed by genetic and/or epigenetic studies and *Pitx2* knockout mice that this gene is required for the proper development of several organs and structures, such as the pituitary gland, craniofacial region, eyes, heart, abdominal viscera and limbs [30,31,32,33,35,36,43,50,55,56,57,58]. In human, *PITX2* was identified by positional cloning of the 4q25 locus in patients with Axenfeld-Rieger syndrome [53]; a syndromic condition coursing with cardiovascular outflow tract malformations, eye dysmorphogenesis, craniofacial and pituitary abnormalities. Furthermore, Cox et al. [52] reported a new PITX2 isoform (*PITX2D*) in humans generated by the *PITX2C* alternative promoter and differential splicing, acting as a dominant negative isoform, specifically against PITX2A and PITX2C (Figure 2). *PITX2* mutations are associated to Axenfeld-Rieger syndrome [53,59,60,61] and several cardiac defects, as we will detail below.

## 4. Pitx2 and Left-Right Signalling

During the early steps of gastrulation, the developing mouse and human embryo are symmetrical. Soon after gastrulation, the first signs of bilateral asymmetry are observed as the developing heart bulges ventrally and then bends to the right. Genetic analyses have demonstrated that morphological asymmetry is preceded by molecular asymmetry [62,63,64], Several molecules have been identified in different species to be asymmetrically expressed during gastrulation such as *lefty-2* in *Xenopus* and mice [65,66,67,68,69,70,71], *Nodal* in *Amphioxus*, *Xenopus*, zebrafish, rat and mice [72,73,74,75,76], *cryptic* in mice [77], *Charon* in zebrafish [78], *Fgf8* and Hh in chicken and mice [79,80], *Cerl-2* in mice [81,82,83,84] and *SnR* in chicken [85]. Importantly, several of these asymmetric regulators display left/right differential expression in the lateral plate mesoderm (LPM), while others already display asymmetric expression in the embryonic midline left/right organizer (LRO), within the developing node. In fact, differential expression in the node [86] and directional flow mediated by cilia in the node [87,88,89,90,91] have been extensively reported to be governing left/right symmetry break. In addition, cell movements within the node have also been implicated in left/right asymmetry [92]. However, two important conclusions can be drawn from all of these datasets; first, the node plays a fundamental role in establishing left/right symmetry break and particularly node cilia and node cilia-related flow (with some exceptions such as the chick and the pig); and second, the left/right symmetric break is a conserved morphogenetic process in most vertebrate phyla having a core signalling cascade represented by *nodal > Pitx2* with different variable players in distinct species (Figure 1). Importantly, *Pitx2* is therefore the last effector of the left/right signalling cascade transmitting positional information from the uncommitted LPM to distinct organ primordia such as the heart, lung and gut, among others [93,94,95], leading to distinct sidedness alterations within these organs if impaired [45,55,96,97,98,99,100].

Thus, the left LPM provides different signals controlling embryonic left-right asymmetry. Several laboratories have shown that Pitx2 expression is confined to left LPM in chick, frog and mouse [30,31,32,33,56,57], as well as the cardiogenic precursors of the left SHF [101]. During development, *Pitx2* continues to be expressed asymmetrically in several organs. Over-expression studies in *Xenopus* and chicken demonstrate that Pitx2 is important in the determination of vertebrate heart and gut looping [30,31,32,33,56,57].

*Pitx2* isoforms distinctly contribute to the regulation of left-right asymmetry [99,102,103]. *Pitx2a*, *Pitx2b* and *Pitx2c* are symmetrically expressed in the head at the beginning of development, but *Pitx2c* is only expressed asymmetrically in the LPM from two to four somite stages in the mouse. During development and adulthood, *Pitx2c* expression still remains in the developing heart [104,105,106] (Figure 2). This expression pattern is highly conserved among species, highlighting the role of Pitx2 during development [7,33,93,94,107,108]. Experimental models of gain and loss of function demonstrated that Pitx2 is dispensable in driving looping directionality in mice [32,33,35,43,58,96,109], contrasting with those findings in other experimental models [30,31,32,33,56,57]. To date, such discrepancies remain to be fully elucidated.

## 5. Genetics of Congenital Heart Diseases

Congenital heart disease (CHD) is the most prevalent developmental abnormality in humans. CHD is indeed the most common non-infectious cause of infant morbidity and mortality. Increasing evidence has demonstrated a crucial contribution of genetic defects in the pathogenesis of CHD. Mutations in several transcription factors such as *HAND1*, *HAND2*, *TBX5*, *GATA4*, *NKX2.5* and *TBX20* have been identified in patients affected with distinct congenital heart diseases, such as atrial septal defects (*TBX5*, *GATA4*, *NKX2.5*, *TBX20*), Tetralogy of Fallot (*TBX5*, *TBX20*), ventricular septal defects (*HAND2*), double outlet right ventricle (*HAND1*), either syndromic (Holt-Oram; *TBX5*) or isolated (see for recent reviews [110,111]). However, CHDs are genetically heterogeneous, and at present, the genetic determinants of CHD in most patients remain yet unknown. We provide herein current state-of-the-art evidence on the contribution of the homeobox transcription factor *PITX2* as a genetic determinant of congenital heart diseases.

## 6. Left-Right Signalling and Congenital Heart Diseases

The adult heart is an asymmetric organ. It is topologically located outside the body midline with the apex oriented to the left. Morphologically, the atrial chambers are rather similar, but conspicuous differences, such as the extent of the smooth region and the abundance of the pectinate muscles, provide hallmarks for distinguishing right and left atrial appendages [6]. Such left/right atrial differences are more easily assessed if their venous connections are taken into account. In humans, four pulmonary veins drain into the left atrium, whereas two caval veins (right superior and inferior caval veins) and the coronary sinus drain into the right atrium. In mice and other rodent species, pulmonary veins drain with a single orifice in the left atrium, whereas three caval veins drain into the right atrium. Atrioventricular valves are also left/right asymmetric, as the mitral valve has two leaflets and the tricuspid valve three. At the arterial pole, including the ventricles, asymmetry is also evident, as the left ventricle is larger than the right ventricle, and the aortic and pulmonary outlets are displaced from the body midline [5,112]; yet, these differences do not reflect in most cases embryonic left/right differences. Importantly, embryonic left/right contribution to the atria and ventricles is markedly different as reported by *Pitx2* expression [4,7,113].

Several cardiac abnormalities have been classically reported as defects of the establishment of the left/right body axis (laterality defects). Right atrial isomerism (RAI) is the most common defect in which the heart is formed by isomeric right atria and impaired venous return connections, displaying in most cases the absence of direct pulmonary vein drainage to the atria and bilateral caval veins connections. In this context, both atria display a right-specific pattern; i.e., morphologically right atrial appendages. Left atrial isomerism (LAI) is rather less frequent, presenting isomeric left atria with the absence of direct caval vein drainage to the atria and bilateral pulmonary vein connections, i.e., right pulmonary vein to the right atrium and left pulmonary veins to the left atrium. Similarly LAI also displays a left-specific pattern in both atria; i.e., with morphologically left atrial appendages. These cardiac alterations can occur with an impaired left/right topology of all other body organs, a condition dubbed *situs inversus*, or not, a condition dubbed *situs ambiguus*. Several lines of experimental evidence demonstrated that impaired left/right signalling underlies RAI and LAI.

The plausible contribution of left/right signalling to other congenital heart defects such as double outlet right ventricle (DORV), double inlet left ventricle (DILV), atrial or atrioventricular septal defects (ASD and AVSD) remains enigmatic, as well as in syndromic congenital heart defects such as Tetralogy of Fallot. Thus, an important question is when is cardiac asymmetry established during embryonic development and which is the particular contribution of left/right cardiac embryonic tissues to the adult heart. We will provide herein state-of-the-art evidence on the plausible role of left/right signaling, with particular emphasis on the homeobox transcription factor *Pitx2*, on the genesis of these cardiac congenital defects.

## 7. Pitx2 and Congenital Heart Diseases

**Figure 3:**
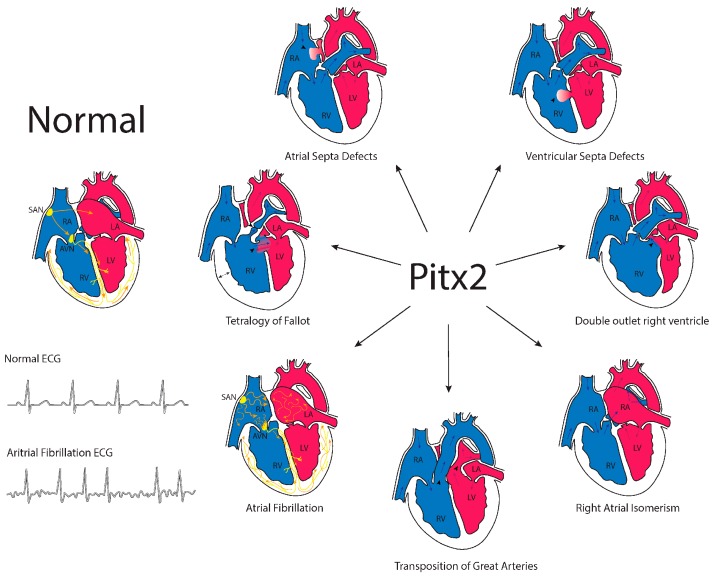
Schematic representation of the contribution of the homeobox transcription factor PITX2 to distinct congenital heart diseases and cardiac arrhythmogenic defects.

Systemic deletion of *Pitx2* in experimental models revealed complex embryonic defects. Abnormal sidedness was observed in distinct developing organs such as the lungs, gut and heart in mice. Embryonic development was compromised at Embryonic Days (ED) 13–14. Within the cardiovascular system, *Pitx2* null mutants displayed right atrial isomerism with impaired venous return [45,55,58]. Pulmonary vein development was severely impaired as was the sinoatrial node development [114,115]. Isoform-specific deletion of *Pitx2c* in mice demonstrated that it was mainly this isoform that was responsible for all of the cardiovascular embryonic defects [116]. Furthermore, conditional deletion in mice highlighted the importance of *Pitx2* driving cardiac left-right asymmetry from the secondary heart field [117] and opened up the possibility of spatio-temporally-controlled deletion of *Pitx2* in distinct cardiac compartments. Tessari et al. [118] conditionally ablated *Pitx2* function in the early embryonic cardiomyocytes, and surprisingly, right/left cardiac asymmetry was unaltered. Atrial and ventricular-specific deletion in mouse embryos [104], as well as temporal deletion in postnatal hearts [118,119], displayed similar findings. Only when *Pitx2* deletion was carried out in the nascent cardiomyocytes, right atrial isomerism was observed [120]. Overall, these data demonstrate a highly controlled temporal and tissue-specific action of Pitx2 during cardiac development that, if impaired, can lead to distinct cardiac congenital heart diseases. Therefore, these observations postulate *Pitx2* as a candidate gene for cardiac congenital heart diseases (Figure 3).

CHARGE syndrome is characterized by ocular Coloboma, congenital Heart defects, Atresia of the choanae, Retarded growth, Genital hypoplasia and Ear anomalies including deafness in the context of global developmental delay. Initial screening in CHARGE syndrome patients discarded *PITX2* as a causing gene [121]. Subsequent screening in a small cohort of transposition of great arteries (TGA) patients similarly failed to identify *PITX2* causal mutations [122]. More recently, *PITX2* point mutations have been identified in distinct isolated cardiac congenital heart diseases [123], such as atrial septal defects (ASD) [124], TGA [125], ventricular septal defect (VSD) [125,126] and Tetralogy of Fallot [127]. A *PITX2* mutation has also been identified in a patient with compound congenital heart disease (CHD), i.e., DORV and VSD. In all cases, functional analyses of their corresponding *PITX2* mutation leads to decreased transcriptional activity and reduced synergistic activation with *NKX2.5* [124,125,126,127] (Table 1). Furthermore, point mutations in *CITED2*, identified in a Tetralogy of Fallot patient with aortic stenosis, impaired *PITX2* and *VEGF* expression [128], further support a functional role for PITX2 in this type of CHD. In addition to the identification of *PITX2* mutations in isolated CHD, a loss-of-function *PITX2* mutation has been identified in an Axenfeld-Rieger syndrome patient carrying cardiac endocardial cushion defects. Such a *PITX2* mutation provides no transcriptional and synergistic activity with Nkx2.5 [129]. Overall, these data demonstrate a pivotal functional role of PITX2 in cardiac congenital heart diseases (Figure 3).

## 8. Beyond CHD: Pitx2 and Atrial Fibrillation

Atrial fibrillation (AF) is the most common cause of arrhythmogenesis, and it has been related to several risk factors, such as advanced age, male gender, hypertension, obesity, ischaemic heart disease, myocardial infarction, valvular diseases and hyperthyroidism [134,135,136].

Several genome-wide association studies (GWAS) have been published reporting chromosomal loci in association with atrial fibrillation (AF). Four different genetic loci on chromosomes 1q21-KCNN3, 4q25-Pitx2, 16q22-ZFHX3 and 16q13-IL6R have been associated with this arrhythmia [137,138,139,140,141]. GWAS meta-analyses have provided additional risk variants associated with AF, implicating six new loci in AF; *CAV1*, *HCN4*, *SYNE2*, *SYNPO2L*, *PRRX1* and *WNT8A* [140,142,143], but importantly, the most relevant associated risk variants are those located in the vicinity of the *PITX2* locus [104,105,106,107,144,145,146,147]. Thus, this seminal GWAS study by Gudbjartsson et al. [137], identifying single nucleotide polymorphisms (SNPs) at the 4q25 locus, proposed an association between PITX2 and AF, as a causative molecular link. Thus, a novel role for Pitx2 in the adult heart has therefore emerged.

Distinct laboratories, including ours, have demonstrated that PITX2 potentially regulates AF through modulation of multiple genes implicated in AF; *KCNN3* [140,145,147]; *TBX5* [148,149,150]; *HCN4* [145,151]; *KCNJ2* [104,152]; *CAV-1* [145,147]; *KCNQ1* [151,153]; *ZFHX3*; *SYNE2*; *Il6R* [147]; ENPEP [154]. In addition, a transcriptional network that linked AF risk loci has been recently described. *Tbx5* directly activated *Pitx2*, and *Tbx5* and *Pitx2* antagonistically regulated membrane effector genes *Scn5a*, *Gja1*, *Ryr2*, *Dsp* and *Atp2a2* [150] (Table 2).

Genomic regions containing the risk variants have been reported to play a role in *Pitx2* regulation in a tissue- and isoform-specific fashion [154,155], and a functional role for Pitx2 in atrial arrhythmias using distinct experimental models has been demonstrated [104,152,156]. Experimental studies have demonstrated that Pitx2 loss of function predisposes to atrial arrhythmogenesis. Mice heterozygous for *Pitx2* are susceptible to AF during programmed stimulation [106]. *Pitx2* haploinsufficiency predisposes to AF in electrically-stimulated adult mice, provoked by ectopic *Shox2* expression in the foetal left atrium (LA), which in turn deregulated other pivotal sino-atrial node genes such as *Hcn4* and *Tbx3* [105].

Other substrates for AF are left-sided electrophysiological defects generated by *Pitx2* impairment in atrium-specific conditional *Pitx2* mouse mutants [104]. In this model, in foetal and adult stages, voltage-gated sodium and inward rectifying potassium channels are abnormally expressed in the atrial myocardium [104]. Moreover, dysregulation of ion channels resulting from impaired *Pitx2* dosage has been reported in adult *Pitx2* heterozygous mutant mice [106]. In *Pitx2c* heterozygous mutant mice, the expression of TWIK-related acid-sensitive K+ channel (TASK-2) and TASK-like background currents are reduced, although I_K1_ is not altered [157]. Impaired gap junction expression in adult heterozygous *Pitx2* mutant mice has been reported [106], being in concordance with the fact that *Pitx2* controls *Gja5* expression [104,152] and with reports of mutations in gap junctional proteins in the context of AF ([158]). Surprisingly, chronic AF patients display upregulation of *PITX2C*, contributing to I_Ks_ increase and I_Ca,L_ reduction [159]. While these controversial findings remain to be elucidated, currently we can conclude that impairing *Pitx2* (upregulation or downregulation) is critical for cardiac electrophysiology and, thus, tightly linked to the onset of cardiac arrhythmias. 

Our laboratory recently reported that *Pitx2* insufficiency also regulates *WNT8* expression, which modulates a complex gene regulatory network, including multiple microRNAs, with a large impact on calcium homeostasis control and pro-arrhythmogenic events [147]. Multiple microRNAs are downstream from *Pitx2* and involved in AF. ChiP-sequencing studies have reported that *Pitx2* positively regulates miR-17-92 and its related homologue miR-106b-25. Deficient mice in these miRNAs clusters show arrhythmia susceptibility and dysregulation of *Shox2* and *Tbx3*, similar characteristics to *Pitx2*-deficient mice [151]. I_K1_ channel expression is modulated by miR-1 [160], which is upregulated in *Pitx2* atrial-deficient mice [104]. A significant number of microRNAs differentially expressed in AF patients are regulated by *Pitx2* and *Wnt* signalling, such as miR-1, miR-26b, miR-29a, miR-106a/b and miR-133 [147,161].

**Figure 4:**
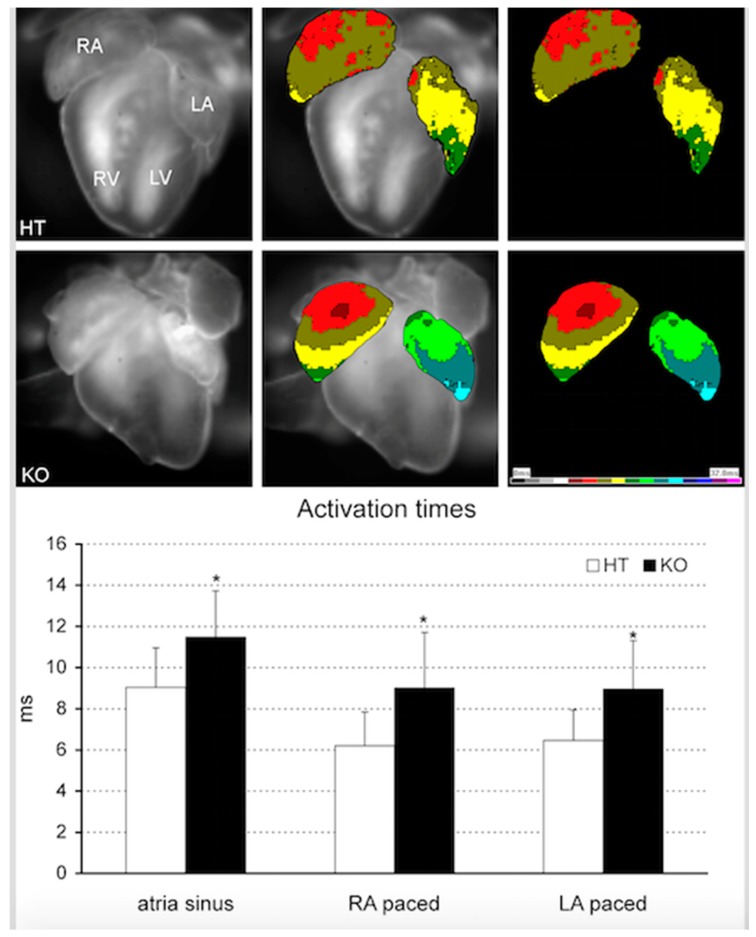
Optical mapping analyses of embryonic heterozygous (HT) and homozygous (KO) atrial-specific Pitx2 mutants. Observe that atrial activation times are significantly slower in homozygous as compared to heterozygous hearts. In addition, right and left atrial pacing, respectively display similarly slower activation times, demonstrating an inherent electrical difference between heterozygous (HT) and homozygous (KO) atrial-specific Pitx2 mutants.

However, a question that remains unsolved is whether impaired *Pitx2* function already alters cardiac function during embryogenesis, as suggested by Wang et al. [105], or whether it is mainly an adulthood deficiency that is causing atrial fibrillation, as suggested by Chinchilla et al. [104]. We demonstrated herein that atrial-specific Pitx2-insufficient embryonic (ED18.5) mice showed arrhythmogenic substrates already at these early stages in homozygous knockout mutants. These animals showed prolonged atrial activation time (Figure 4) under the sinus rhythm. Similarly, slower impulse propagation was noted also in the electrically-stimulated beats, both in the left and right atrial appendages (Figure 4). Interestingly, there was no significant morphological substrate present at that point, as the amount of collagen, detected by Picrosirius red staining, was uniformly low and not different between controls and mutants. Furthermore, connexin43 expression in the atria did not differ between controls and mutants. These results therefore suggest that functional changes in atrial-specific Pitx2-insufficient mice pre-date structural differences. We therefore propose herein a reconciling hypothesis. Pitx2 insufficiency already predisposes from embryonic stages to atrial electrical impairment in the absence of structural alterations. Onset of spontaneous arrhythmogenic events might be triggered by additional AF risk factors such atrial dilation [104] or by progressive Pitx2 downregulation that can course with age and hypertension [162]. Furthermore, the first arrhythmogenetic events also lead to Pitx2 downregulation, subsequently predisposing to the onset of additional arrhythmogenetic events [156].

## 9. Conclusions and Future Perspectives

Congenital heart diseases are multifactorial. Over the last few decades, we have witnessed a large advance in the understanding of the genetics of congenital heart diseases. Mutations in different transcription factors have been identified in familial and sporadic cases of distinct congenital heart diseases. Genetic engineering in mice has further provided additional evidence of the functional role of these transcription factors (and their corresponding mutations) in CHDs. Herein, we have provided a state-of-the-art review of the contribution of the homeobox transcription factor *Pitx2* to congenital heart diseases. *Pitx2* participates in early embryonic left/right signalling and cardiac embryogenesis, and it remains to be expressed in the adult heart. Therefore, the contribution of *Pitx2* to CHDs might involve distinct roles at different developmental stages. In this line of thinking, Fakhro et al. [163] reported a genetic screening of rare copy number variations in heterotaxia patients and identified genes involved in cilia function. Experimental manipulation of these cilia-related genes led to impaired *Pitx2* expression in *Xenopus* experimental models, providing functional links between cilia, left/right, *Pitx2* and heterotaxia. Distinct genetic *Pitx2* manipulations have also reported distinct congenital heart diseases, establishing temporal and tissue-specific contributions [117,118,120]. More recently, a link between *Pitx2* and atrial fibrillation has been reported [164,165,166]. Our data and others demonstrated that electrical alterations underlying *Pitx2* insufficiency are already present at foetal stages, suggesting therefore a congenital substrate on the future onset of atrial arrhythmogenesis. Thus, whether AF might be considered as a developmental disease should be debated. 

In addition to experimental models demonstrating the functional role of *Pitx2* in CHDs, emerging evidence also identified point mutations associated with distinct CHDs, ranging from isolated atrial septal defects to complex cases of Tetralogy of Fallot. At present, evidence of the functional role of these point mutations in these CHDs is rudimentary. CRISPR/Cas9 gene editing of these mutations might in future shed light on the specific contribution of *Pitx2* mutations to CHDs.

## Figures and Tables

**Table 1:** Summary of the distinct cardiac congenital heart diseases associated to Pitx2.

Congenital Heart Disease	Type	References
**Atrial Septa Defect (ASD)**	Ostium primum ASD Ostium secundum ASD Patent foramen ovale Sinus venosus ASD Common or single atrium	[124] [130,131,132]
**Ventricular Septa Defect (VSD)**	Perimembranous/membranous VSD (type 2) Inlet and AV canal VSD (type 3) Left ventricular to right atrial communication (Gerbode type)	[125] [126]
**Double Outlet Right Ventricle (DORV)**	DORV with subaortic VSD DORV with subpulmonic VSD	[7,10] [38,39]
**Right Atrial Isomerism (RAI)**		[27,28] [133]
**Transposition of Great Arteries (TGA)**		[125]
**Tetralogy of Fallot (TOF)**		[131]

**Table 2:** Summary of distinct signalling pathways regulated by Pitx2 and currently associated with atrial fibrillation.

Cardiac Disease	Type	Related Pathways	References
**Atrial Fibrillation (AF)**	Paroxysmal Persistent Permanent	Pitx2 regulates AF through modulation of multiple genes and miRNAs implicated: → KCNN3, TBX5, HCN4, KCNJ2, CAV-1, KCNQ1, ZFHX3, SYNE2, IL6R, ENPEP, SCN5A, GJA1, RYR2, DSP, ATP2A2, SHOX2, TBX3, WNT8A → miR17-92, miR106b-25, miR-1, miR-26b, miR-29a, miR-106a/b, miR-13	[104,105,106] [137,138,139,140,141,142,143] [145,146,147] [154] [147]
